# Nutrition practices and race experiences of ultra-marathon athletes in the marathon des sables, a qualitative analysis

**DOI:** 10.3389/fspor.2026.1791616

**Published:** 2026-06-18

**Authors:** Tansy Ryan, Ed Daly, Lisa Ryan

**Affiliations:** Department of Sport, Exercise & Nutrition, School of Health, Sport Science & Nutrition Atlantic Technological University (ATU), Galway, Ireland

**Keywords:** behaviour, gastrointestinal symptoms, marathon des sables, nutrition, psychological effects, ultra-endurance, ultra-marathon

## Abstract

**Introduction:**

Participation in ultra-endurance running events, or ultramarathons, has grown substantially in recent decades. These events often take place in challenging environments, such as deserts with extreme heat, which place considerable physiological and psychological stress on participants. Combined with high training demands and limited access to nutrition and hydration, such activity can have short- or long-term health consequences.

**Objective:**

To qualitatively examine the nutrition practices and experiences of five ultra-endurance runners in their preparation for, participation in, and recovery from the self-sufficient multi-day ultra-marathon situated in the Sahara Desert (the Marathon des Sables) to support professionals in their understanding of such experiences and, ultimately, their development of appropriately supportive recommendations.

**Methods:**

This qualitative study used semi-structured interviews (*n* = 3 per participant) and reflexive thematic analysis to examine the experiences of five ultra-endurance runners. The resulting transcripts were thematically analysed using Braun and Clarke's framework to identify common practices in nutrition and training approaches.

**Results:**

Common dietary practices included a reliance on unprocessed foods before the event and dehydrated foods during the event, while the recovery period was much more relaxed and lacked any specific dietary approach. Gastrointestinal symptoms varied between participants, ranging from mild discomfort to nausea and vomiting which impacted race performance. Though not an initial aim of this research, it became apparent that ultra-endurance running is accompanied by extreme psychological challenges which impacted race performance, highlighting the interaction between nutritional practices and psychological responses during the event. Discussion: Findings from this exploratory study suggest that while commonalities exist in both the physiological and psychological experiences of ultra-endurance runners, there remains a high degree of diversity in athletes' experiences. This highlights the need for further research and collaborations across disciplines to develop a holistic approach to support optimal ultra-endurance preparation, performance, and recovery.

## Introduction

Ultra-marathon running, a term oftentimes used interchangeably with ultra-endurance running or ultra-running, encompasses foot races defined either by distance, being more than the typical marathon length of 42.2 km, or by a time limit. Historically, the latter has been characterised as a single bout of exercise lasting over four hours. However, this benchmark has more recently been revised to over six hours due to the surge in this sport's popularity (1). Ultra-endurance running events, known as ultramarathons, have seen a global increase in participation of over 1,600% in recent decades, from approximately 34,401 total yearly participants in 1996 to 611,098 in 2018 (2). However, this increase in participation does not coincide with a general increase in performance, with contemporary ultra-endurance runners now generally older and slower than those in previous years. In their investigation of participation and performance trends of 12- and 24 h events from 1876 to 2020, Thuany et al. (3) highlighted that the lowest mean age was observed for female runners in 12 h events (43.46 ± 10.16 years), while the highest mean age was for male runners in 24 h events (46.13 ± 10.83 years). They noted that for all groups except females in 12 h events, the mean age was above 40 years old. This growth is a result of factors such as an increased number of events (growing from 379 100 km events in 2000–2009 to 1373 in 2010–2019), and more women and people over 80 years old participating. In relation to performance, in analysing the trends of 2,067 100 km ultra-marathons globally from 1960 to 2019, Stöhr et al. (4) found overall racing performance significantly (*p* < 0.001) increased up to the decade 1990–1999, with no further significant (*p* > 0.05) differences afterwards. Taken together, these trends indicate that while ultra-endurance running has become increasingly popular, improvements in performance have plateaued, suggesting that many participants are competing at amateur rather than elite level. This growing participation, combined with the demanding nature of this sport, underscores the need to better understand the physiological and psychological impacts of participating and to identify how best to support athletes in managing these demands.

Many ultra-endurance runners are considered amateurs. That is, there is a growing number of ultra-endurance runners who are older and slower than those placing highly in events. These amateur athletes often have lower nutrition knowledge than their elite counterparts, resulting in dietary strategies which are suboptimal and leave individuals at a heightened risk of injury (5–7). Though short-distance recreational running is widely recognised for its association with a myriad of health benefits, with as little as one year of habitual running (mean ± SD: 2.3 ± 1.0 h/week) significantly improving cardiorespiratory fitness, ultra-endurance running may have contrary effects (8, 9). Physiological repercussions, though often transient, are multifaceted and may include negative impacts on the musculoskeletal system, cardiac biomarkers, organ functioning, and immune responses. Musculoskeletal damage includes issues such as overuse injuries, stress fractures, and inflammation, often as a result of high training volumes. An increase in cardiac biomarkers such as creatine kinase, creatine kinase-MB, cardiac troponin I, N-terminal pro-brain natriuretic peptides, and C-reactive protein may increase the likelihood of heart damage. Additionally, various organs and organ systems, such as the skeletal system, liver, and kidneys, experience disruptions to biomarkers indicating a pathological process. Moreover, ultra-endurance running has been shown to lead to an acute inflammatory response with an increased number of an array of inflammatory markers. As a result, ultra-endurance runners are often at an increased risk of infection, particularly respiratory, after an event (7). A typical physical consequence of ultra-endurance running is disturbance to the gastrointestinal tract, which may manifest as gastrointestinal symptoms (GIS) such as nausea, dizziness, vomiting, abdominal pain, or changes in bowel movements. These symptoms can subsequently harm exercise performance (10, 11). Though these GIS are typically transient, there remains the potential for them to evolve into more serious, long-term health complications (12, 13).

In addition to its physiological effects, ultra-running entails significant mental challenges, with psychological factors often pivotal in an athlete's racing performance and ultimately their event completion (14, 15). Previous qualitative investigations into the psychological aspects of ultra-endurance running have recruited individuals with experience in various race types, such as track ultra-marathons, 24 hour races, and multi-day events (16). Such research has suggested that while similarities exist between ultra-endurance athletes' psychological identity, each athlete's journey into such sports is unique (17). Moreover, literature focusing specifically on the motivations of ultra-endurance runners emphasises that due to the complexity of this sport, there is a necessity for individualised training and support strategies to promote an athlete's success (18).

Current nutrition recommendations for ultra-endurance runners' training and racing have been defined by the International Society of Sports Nutrition Position Statement. Though specific macronutrient recommendations are defined in this statement (for racing: 150–400 kcal.hr for <50 mile events or 200–400 kcal.hr for >50 mile events, 30–50 g.hr carbohydrates, 5–10 g.hr protein, 1.1–1.7 g.hr fat, 450–750 mL.hr hydration, >575 mg.L sodium), the overarching recommendation is that athletes allow ample time in training to trial and alter nutrition, hydration, and GIS management strategies. These individualised approaches should aim to avoid caloric deficits, macronutrient deficiencies, dehydration, and electrolyte imbalances while simultaneously avoiding or alleviating GIS, and remaining suited to an individual's taste and preferences (19). However, it is essential to note that these guidelines are aimed at ultra-endurance runners participating in single- rather than multi-stage events (20). Furthermore, the vast majority of existing literature concerning ultra-endurance running has concentrated on single-day events, eliciting results and recommendations which may not necessarily be transferable to multi-stage event participants (21). A finding of significance, though, is that single-stage participants regularly fail to meet dietary recommendations, and, as multi-stage events are more demanding, there is a high likelihood that this issue also exists for multi-stage competitors (22).

Amongst the most popular of ultra-endurance running challenges is the Marathon des Sables (MdS), a 250 km, six-day, multi-stage ultra-marathon. Situated in the Moroccan desert, participants contend with harsh, sandy terrain, minimal shade, and peak daytime temperatures of over 40°C (23). Echoing the broader trend in ultra-endurance running, participation in this event has surged in recent decades, from 192 finishers in 1995 to 848 in 2025 (24). Despite this, there is a sparsity of research examining the lived experiences of individuals partaking in this unique race (25, 26). The existing literature has predominantly employed quantitative methodologies to assess the impacts of participating in this event, such as its effect on energy balance, heart rate, or running speed (25–29). For instance, Ioannou et al. (27) reported a mean heart rate of 142 ± 10 bpm and an average running speed of 5.2 ± 0.8 km.hr during the MdS. Hough and Earle (28) found while investigating the nutrition of an MdS participant, a total daily energy intake of 2,946 ± 358 kcal but an expenditure of 3,006 ± 1,030 kcal, resulting in a total deficit of 9,609 kcal over the duration of the event. In parallel, investigations into the psychological facets of this race have focused on quantitative data derived from psychological questionnaires. Though these have yielded valuable insights, they have also highlighted the need for a deeper exploration into the individual experiences of MdS participants (29–32).

The scarcity of research employing qualitative methodologies to understand the experiences of MdS participants impedes the development of evidence-based recommendations and guidelines, subsequently compromising athletes' ability to sufficiently physiologically or psychologically prepare, perform, and recover from such ultramarathons (33). This can further manifest into short- and long-term issues for athletes' health and performance. Additionally, research on marathon runners cannot necessarily be directly extrapolated to develop guidelines for ultra-endurance runners, as these cohorts have been shown to have significantly different anthropometric, training, and periodisation characteristics (34). Moreover, there is a notable absence of research investigating the difference in nutrition and hydration strategies between finishers and non-finishers of ultramarathons (35). Despite the growing body of research in this area, there remains limited understanding of how these athletes experience and navigate the demands of multi-stage ultra-endurance events such as the MdS, particularly how these factors interact in real-world settings. This interaction between physical strain and psychological responses has been described in models of endurance performance, such as the psychobiological model of fatigue, which highlights the role of perceived effort and cognitive factors (36). Recognising the importance of evidence-based literature in developing recommendations that adequately support ultra-endurance runners, this study aimed to qualitatively explore the nutrition practices and lived experiences of individuals participating in the MdS ultramarathon, including the interaction between nutritional and psychological demands experienced throughout the event.

## Materials and methods

This research utilised qualitative analysis, whereby the lead author (TR) individually interviewed participants on three separate occasions.

### Ethics and procedure

The research received ethical approval from the Research Sub-Committee of the Academic Council of Atlantic Technological University (ATU_01032024_TR). Participants were recruited using a purposive sampling approach, specifically targeting individuals preparing for and participating in the MdS. For participant recruitment, an infographic outlining the nature of this research, the eligibility criteria necessary to participate, and the lead researchers' contact details was developed. The infographic was distributed to running clubs and organisations, individuals with large numbers of followers online on Facebook and Instagram whose posts often relate to ultra-endurance running, and individuals who had previously posted about their plan to partake in the MdS in 2024.

Eligibility criteria consisted of being ≥18 years of age, healthy with no current diagnosed health conditions, and having been training for and planning to participate in the MdS. Individuals who contacted the research team were sent a participant information sheet offering more in-depth details on the investigation, such as what partaking entails and the confidential nature of the data gathered. Before beginning the interview process, informed consent was obtained from each participant. All of the information used throughout this study has been anonymised to protect the participants from possibly being identified. A total of *n* = 5 participants were recruited with a 100% follow-up rate.

### Data collection

As part of the ethics application process, the research team defined and agreed upon a series of overarching questions before conducting the interviews with participants. Each participant was interviewed virtually via Microsoft Teams three times (within 1–2 weeks prior to the event, within 2–3 days prior to the event, and within 1 week after the event) by the lead researcher (TR). Each interview was approximately 10–20 min long (mean ± SD: 15 ± 5 min). This length was chosen as the interviews took place during hectic periods when athletes were either preparing for, travelling to/from, or recovering from the event. The audio recordings of each interview were transcribed verbatim into Microsoft Word. These interviews were semi-structured in their design, and though they mostly remained in line with the previously developed interview questions, further probing with additional questions was employed as and when necessary to draw out more information from the participants. The questions were based upon a previously developed and validated questionnaire for ultra-endurance runners developed by Scrivin et al. (37) (Table 1). The interview guide covered key topics including nutrition practices, training routines, race preparation, in-race nutrition and hydration strategies, gastrointestinal symptoms, and recovery practices. Triangulation was achieved by interviewing multiple individuals at three different timepoints during their MdS race experience. While the interviews were relatively short (10–20 min), they were carried out at three key timepoints (before the event, immediately before, and after), which allowed for a broader and more reflective understanding of participants' experiences over time. This meant that, although each interview was brief, together they captured changes in participants' perspectives across preparation, participation, and recovery. The semi-structured format also allowed for flexibility, and follow-up questions were used where needed to encourage participants to expand on or clarify their responses. This helped to ensure that, despite the time constraints surrounding the event, the data collected was sufficiently detailed to support meaningful analysis.

**Table 1:** An example of questions used during interviewing athletes to explore the nutrition approach and gastrointestinal symptom management strategies they plan to execute during the marathon des sables, and potential prompts used for further extracting information.

Question	Prompts
Can you describe for me, how you are going to approach the upcoming event in terms of nutrition?	(a) What are the main foods, fluids and supplements you will be ingesting? (b) Do you have a nutrition (food, fluid, supplements) plan? (c) Will you be tracking your nutrition? (d) Do you give any consideration to the times at which you ingest these? (e) How, if at all, does your nutrition change based on the environment you’re racing in?
Are you expecting to experience any gastrointestinal symptoms during the event?	(a) At what point are you expecting to experience these? (b) Which symptoms are you expecting to experience? (c) How do you plan to manage these symptoms?

The resulting transcripts were then reviewed against the original audio recordings to ensure accuracy. The first interview was conducted 1–2 weeks before the MdS race, with discussions on participants' general eating behaviours and training routines. The second interview, scheduled to take place within the week before the MdS, involved discussions around participants' approach to this specific event, what, if any, changes were implemented to support them in preparation for the event, and their expectations for the event (i.e., the amount and timing of their eating and hydrating, their expectations of experiencing GIS, and, if relevant, the strategies they intended to employ to alleviate GIS. The third and final interviews took place within the first week after the MdS. This interview focused on gaining an insight into participants' experiences throughout the MdS event, whether the event went as they had expected, what, if any, unexpected challenges arose, how these challenges were managed, and how participants have approached the recovery period.

### Data analysis

The resulting data from interviewing participants was qualitatively analysed per the Braun and Clarke (38, 39) methodology, a framework consisting of six key phases. This process involved the lead researcher conducting an initial reading and re-reading of the transcripts to familiarise herself with the data, noting initial observations and potential patterns (phase 1—familiarisation). Following this, succinct labels or codes were generated that captured the essential interview data (phase 2—coding). These initial codes were then examined to identify patterns in participants' broader experiences, behaviours, and nutrition practices (phase 3—theme generation). The themes initially developed were reviewed and checked against the data to ensure they accurately portrayed its information (phase 4—theme development and review). Each theme identified was given a name to reflect its contents and represent it accurately (phase 5—theme refinement and naming). Finally, the themes were discussed with the inclusion of relevant data extracts (phase 6—writing).

Core themes identified included Balancing Control and Flexibility in Everyday Practices, Managing the Desert: Nutrition, Hydration, and Gastrointestinal Strain, Recovery as Release and Reset, and Mental Battles in the Sahara. Although psychological experiences were not an explicit focus of the initial research aim, they emerged consistently during the coding process. In line with reflexive thematic analysis, themes were developed inductively from the data rather than being defined in advance. As analysis progressed, psychological challenges were identified across multiple participants, including cognitive fatigue, distress, and coping strategies. Initially, eight codes were generated from analysing the transcripts to capturing key concepts relating to participants' nutrition practices, their experiences of their MdS journey, and challenges they faced. These codes were grouped into broader themes through an iterative process. This was performed by the lead researcher (TR) and reviewed by the other researchers (ED and LR). Any discrepancies were resolved through group discussion. Themes were developed inductively from the data, allowing patterns to emerge naturally from participants' accounts while also highlighting areas of variation between individual experiences. Several practices were implemented to support the credibility of this study. In line with good practice and qualitative research, the authors recognised the role of the lead researcher's positionality in shaping the research process and interpretation of the data. This lead researcher is a qualified nutritionist and has previous experience researching the population. This position facilitated a strong understanding of the nutritional and physiological concepts discussed. The co-authors also have extensive experience in nutrition and qualitative research and acted as critical peers by reviewing the codes and themes generated, challenging potential biases, and supporting reflexivity. The lead researcher kept a journal documenting the progress of this study, information on its activities, and reflective notes, and the research team held regular meetings to discuss the progress of this investigation, reflect on the data, and discuss findings. These practices helped to ensure reflexivity and foster rigour in the research process. To minimise potential bias, coding and theme development were discussed among the research team, with interpretations reviewed and refined through regular meetings. This process supported critical reflection and helped ensure that themes were grounded in the data.

## Results

The present study aimed to qualitatively analyse the nutrition practices and experiences of five individuals participating in the MdS ultramarathon. A total of *n* = 5 participants were recruited for this investigation (*n* = 4 male). The sample size is consistent with qualitative research employing in-depth, longitudinal interview designs within niche populations, where emphasis is placed on the richness and relevance of the data obtained rather than sample size alone. In line with the concept of information power (40), the narrow study aim, specificity of the sample (participants preparing for and competing in the Marathon des Sables), and the depth of data collected were considered sufficient to address the research question. Additionally, the longitudinal design, incorporating three interviews per participant (15 interviews in total), enhanced data richness and enabled the development of nuanced and meaningful themes. During analysis, patterns were consistently identified across participants, indicating sufficient depth and coherence within the dataset to support thematic development. Information on the participant cohort is outlined in Table 2. Table 3 outlines the beverages reported by participants, which commonly served as sources of energy, carbohydrates, electrolytes, and/or salt. One participant (P5) reported her weekly training commitment in miles per week, as opposed to hours per week. Therefore, this data was omitted when calculating this variable's mean and standard deviation. Through analysing the resulting transcripts, four overarching themes became evident. These were 1) Balancing Control and Flexibility in Everyday Practice, 2) Managing the Desert: Nutrition, Hydration, and Gastrointestinal Strain, 3) Mental Battles in the Sahara, and 4) Recovery as Release and Reset.

**Table 2:** Table outlining the demographic information of participants of this study, as well as their ultra-endurance running experience and weekly commitments to training (with the exclusion of the week prior to an event, in which participants dramatically decreased their training in preparation for an event).

Participant number	Gender	Age (years)	Ultra-endurance running experience (years)	Weekly training commitment (hours per week)	Self-reported as being competitive
1	M	43	5	10–13	Yes, very—there to “compete not complete”
2	M	36	2	10–14	Yes—wants to finish in the top 100
3	M	36	9	10–24	Yes, extremely—on international team, aiming to place highly
4	M	34	1	4–6	Somewhat—generally yes, but not for this event
5	F	55	5	60–100^a^	Somewhat—competitive with herself
Mean ± SD		**41 ± 9**	**4 ± 3**	**11 ± 5**	

a Indicates a response reported in miles, rather than hours, per week of training for an event. For this reason, this data was omitted when calculating the mean and standard deviation (SD).

Bold indicated total values.

**Table 3:** Table outlining participants self-reported nutrition knowledge on a scale from 1 (very little knowledge) to 10 (extremely knowledgeable), their sources of nutrition information, whether they had previously experienced gastrointestinal symptoms (GIS) during exercising and why they believed these occurred, and the strategies they employ to manage GIS.

Participant number	Self-reported knowledge (/10)	Nutrition information sources	Previously experienced GIS	GIS management strategies
1	6/7	Dietician (formal), social media	Yes, mainly in latter stages of races	None, “powering through”
2	6/7	Nutritionists (formal and informal)	Rarely, as result of high sport gels consumption	Avoids: fizzy drinks, heavy reliance on sports gels
3	8	Reading articles online, other athletes, trial and error	Yes, particularly in races with high altitude or after 8–9 hrs of running	Avoids: vegetables, fibre, gluten and lactose
4	7/8	Nutritionist (informal), social media, online articles	Yes, as result of high sport gels or sugar consumption	Avoids: fibre, sugar, heavy reliance on gels.
5	6/7	Trial and error, nutritionist (informal), no longer seeks out new information	Yes, as a result of sport gels	Avoids: sports gels

### Balancing control and flexibility in everyday practices

This theme reflects how participants navigated a tension between control and flexibility, where efforts to optimise performance through structured nutrition and training were often balanced against the need to reduce psychological stress. The participants of this research described experiencing a tension between maintaining strict control over their nutrition and training and allowing themselves flexibility to reduce psychological strain. However, efforts to find this balance often led to counteractive results. This not only shaped their everyday approaches to training and nutrition, but also their social interactions. This was particularly noticeable as one athlete purposefully limited social interactions to a small circle of family and close peers in the lead-up to an event. This was done to minimise exposure to pathogens that could potentially compromise her health and impede her event performance.

P5: “I've stopped going to the gym now because I don’t want to pick up any germs off anybody, so I have no social contacts apart from family from three weeks out of a race like this, I don't see anybody. I don't go in any pubs, restaurants … nothing … that all stops”.

A number of participants recounted previously becoming fixated on their nutritional intake, which subsequently led to a source of stress in their lives. P5 reported previously engaging in fad diets and restricting overall calorie intake, usually in an attempt to lose weight.

P5: “No, no, I've. I've done that. No, I've done that because I did the keto diet”.

Similarly, another athlete (P3) reported eliminating entire food groups, such as those with gluten or lactose, in an effort to improve performance by avoiding GIS.

P3: “I try to avoid gluten before a race normally… I normally avoid lactose foods”.

More recently, participants had “clued up” (P5) and distanced themselves from focusing on calorie tracking after recognising that this behaviour, akin to partaking in “fad” diets, had become a point of stress in their lives and fostered a slightly unhealthy relationship with their dietary intakes, as they had “tried and failed several approaches” (P3). Participants’ dietary habits followed similar patterns, with daily food choices oftentimes mirroring one another. Participants predominantly focused on consuming what they considered to be “clean” foods. These were minimally processed and lacking in added sugars, preservatives, and artificial colours and flavourings. Instead, they were continuously opting for low-processed products and avoiding highly processed products laden with added sugars, unhealthy fats, additives, or preservatives. This may reflect how sometimes efforts to control nutrition practices could become a source of psychological stress, highlighting a tension between performance optimisation and overall wellbeing. Participants described a routine lifestyle whereby their training and diet schedules, though lacking formal structure, followed a loosely defined regime. For example, participants generally completed their longest training sessions on the weekends, correspondingly increasing their carbohydrate consumption in preparation for and to enhance performance during such sessions. Similarly, athletes regularly reported eating “more carb-based food after the runs” (P2) to replenish their glycogen stores and support their recovery. A lack of a formal, structured approach to nutrition may be due to a feeling that they “probably know the general gist, but it's actually how to apply that in practice” that is a challenge (P1).

Through self-experimentation with their dietary intakes, all participants were now at a stage at which they had significantly reduced how often they experienced GIS, which now occurred during “racing more so … training not so much” (P1). GIS are notorious in ultra-endurance running, and with their consequential impact on performance, participants had a shared goal to identify dietary patterns that supported their performance without causing GIS. All participants involved in this research reported either experiencing GIS themselves or witnessing another athlete suffering from severe GIS issues during ultra-endurance running. The GIS experienced by participants during events significantly varied. These could be categorised into upper and lower GIS. The former encompasses symptoms such as headaches, nausea, and dizziness, while the latter comprises abdominal cramping, bloating, vomiting, and disruption to bowel movements. As per the participants' descriptions, these were often related to an overconsumption of energy gels, a change in salt levels and hydration status, or a change in appetite, which resulted in deviance from their race-day diet strategies.

Participants' degree of flexibility in their training and nutrition approaches varied. This was evident as some participants were not concerned about their placement in the event. In contrast, others were clear that they were there to compete and hopefully place highly, terming themselves “a compete-er, not a completer” (P1). This variation in flexibility was also seen in their dietary practices and general daily schedules leading up to an event. Some participants reported a broad approach whereby they solely focused on ensuring an adequate energy intake by increasing meal sizes or frequency.

P3: “for this race particularly, I haven't changed a lot of my diet because it's rather consistent throughout the year anyway, but it's just more so a little bit more carb heavy in the evening than I'm used to … So my day still looks rather similar, just more food in the evening than in the past”.

Comparably, other participants had meticulously prepared and practiced their race-day routine.

P2: “I have done already like, so I've done … basically like two full weeks of like nutrition prep where I've eaten everything that I would have on the race day. So, like from say stage one right there through to stage six like what I would eat in that week just to see what it would be like”.

P3: “So I would plan at the start. so for, I would plan out how much … when my big runs are and like when my big sessions are that I need sort of additional fuelling and then I would plan out like a menu sort of at the start of each week and then make sure I have all my sort of main meals prepped”.

Collectively these findings illustrate how ultra-endurance runners navigate the balance between control and flexibility in their everyday practices and event preparation. While strict rules can enhance perceived readiness, they are often accompanied by psychological stress, which may have a counteractive effect.

### Managing the desert: nutrition, hydration, and gastrointestinal strain

This theme highlights how an extreme environment, in this instance a desert, may shape both nutritional behaviours and psychological responses, with participants continually adapting their intake in response to gastrointestinal discomfort, fatigue, and environmental stressors. The second round of interviews with participants focused on their preparation and anticipated outcomes for the MdS event. Following this, the initial segment of the third and final round of interviews prompted participants to reflect on their experiences of the race. Participants' accounts of the event revealed that nutrition was not only a matter of food choice and preference but also a survival strategy throughout this period. Their in-race experiences highlight how the desert environment shaped their dietary practices, hydration strategies, and GIS management.

In the lead up to the event, participants emphasised the importance of preparation and planning of their food and hydration strategies. A commonality among participants in this research was their reliance on what they termed “clean”, dehydrated foods throughout the duration of the MdS event. Given that this event is self-sufficient, whereby participants are required to carry their belongings throughout the multi-day event, each participant predominantly relied on such food products to obtain a sufficient caloric intake while ensuring a minimal impact on the weights (their food supply and race equipment) they were obligated to carry. Selecting foods that were both energy-dense and lightweight was a key factor shaping participants' dietary choices.

P4: “I'll be eating nut bars as well. I have two of them packed and one small carb gel that I'm still debating if I want to carry”.

Frequently mentioned food items included nuts, seeds, energy bars and gels, pre-made freeze-dried meals by a brand called Expedition Foods, and powdered nutritional beverages. These beverages were often a source of energy, carbohydrates, electrolytes, and/or salt (Table 4).

**Table 4:** Table outlining participants reported nutrition approach for the Marathon des Sables (MdS) event, the foods and supplements they consumed prior to, during, and after the event, and their experiencing of gastrointestinal symptoms (GIS) during the event.

Participant number	Nutrition strategies for the MdS	Foods consumed (pre-MdS)	Foods consumed (generally and/or during-MdS)	Foods consumed (post-MdS)	Supplements used in the MdS	GIS in MdS
1	**Pre-MdS: *Carbohydrate loading***—Increased carbohydrate intake (aiming for approximately 5 g.kg body weight) in the days leading up to the race.	Bananas, bagels, energy drinks, energy bar, cereals, rice	Freeze-dried meals, race nutrition powders, muesli, granola, olive oil, peanut butter, energy bars, stock cubes, cocoa pops, milk powder	Recovery shake, pizza	Salt tablets	Yes—nausea, general discomfort, vomiting
	**During-MdS: *Hydration monitoring***—Using urine color as indicator of hydration status					
2	**Pre-MdS: *Carbohydrate loading***—Increased general carbohydrate intake in the days leading up to the race.	Porridge, gammon, bacon, potatoes, chicken, rice, nuts, dried fruit, energy bars, brioche bread	Freeze-dried meals, stock cubes	Rice, couscous, various proteins	Salt tablets, multivitamins, electrolytes, protein powder	Yes—stomach upset, constipation
	***Trialing*—**Underwent a “mock week” where he trialled his nutrition plan for the event					
	**During-MdS: *Intake target*—**Daily goal of 2,000 to 3,000 kcal each day					
3	**Pre-MdS: Trialing—**Trialed various carbohydrate-rich foods to find ones most tolerated	Rice, couscous, pasta, waffles, porridge, chicken, eggs, peanut butter	Dehydrated foods, stock cubes, carbohydrate drinks	None specified	Salt tablets, protein powder, collagen, carbohydrate drinks	None
	**During-MdS: Intake target—**Hourly carbohydrate goals of >55 g					
4	**Pre-MdS: *Acclimatization*—**Regular sauna use with incremental time increases	Eggs, chicken, fruits (avoids high fibre fruits), cottage cheese, bananas, peanut butter, milk, nut bars	Dehydrated foods, stock cubes, nut bars	Protein powder, recovery shakes, crisps, cookies	Salt tables, multivitamins, omega-3, magnesium, creatine, electrolyte tablets, protein powder, caffeine	Yes—slight discomfort, lack of appetite
	**During-MdS: *Intake target*—**Aimed for salt and electrolytes consumption every 20 min, and for some carbohydrate intake (usually as a gel) every 10–12 km					
5	**Pre-MdS: *Trialing*—**Mimicked the nutrition strategy in long training sessions approaching event	None specified	Salted peanuts, mini cheddar crackers, oat bars, dried fruit bars, jellies, cola, soft cheese, breadsticks, oranges, dates, tins of tuna, cheese slices, dehydrated foods, stock cubes	Recovery shake	Caffeine tablets, hydration tablets	Yes—extreme discomfort, nausea, vomiting
	***Acclimatization—***Began using the sauna in weeks prior to event					
	**During-MdS: *Intake target*—**Set nutrition (specific snack items to be eaten by next checkpoint) and hydration (approximately 500 mL.hour) targets for each checkpoint in the event					

Participants emphasised the importance of relying on food they had previously implemented into their nutrition strategies or experimented with to avoid developing GIS during the event, noting “this is not the time to be trying new things” (P4). As the MdS is held in a relatively hot climate, all participants were acutely aware of the importance of monitoring their salt and hydration levels. With recent changes to MdS management, stock cubes became an essential item to be packed by all participants at this year's event. Although there was a shared initial hesitation around using these stock cubes, they ultimately garnered positive feedback from participants as they provided crucial salt while also introducing new flavours and textures to the participants' otherwise monotonous, repetitive diets.

P4: “and we had the broth cubes … They actually were helpful on the long stage because you get sick of eating salt tablets and sugary like drink which is the electrolytes. So then in the evening I changed to broad cubes. So I would incorporate them even if it would not be mandatory if I had to do it again”.

Amongst the five individuals who participated in this study, three reported experiencing gastrointestinal discomfort to the extent that it impacted their racing performance. For one participant, constipation was the primary GIS they experienced. This was particularly prevalent during the earlier stages of the event, with the participants attributing it to a combination of pre-event anxiety, their recent environmental changes, and the alteration of their dietary intake from their usual food sources to a reliance on dried meals. Fortunately, this issue lessened as the week progressed.

For another participant, despite her deliberate avoidance of social interactions prior to the event, she fell ill early due to stomach upset. This was accompanied by nausea, headaches, dizziness, abdominal cramping, and ultimately vomiting. Early in the day, she noticed that she “didn't have a massive appetite” and was only eating “50% of what I'd taken out, I ate half of each thing I'd taken, you know, kind of really nibbled on it” (P5). As the day progressed, her condition worsened, and she was ultimately forced to stop running to vomit. She vomited an entire, undissolved salt tablet. As a result of this episode, the participant expressed a newfound hesitance to using salt tablets in future races and, though initially reluctant to use them, noted that in the future she plans to experiment more with broth cubes as an alternative source of salt. After vomiting this salt tablet, her GIS partly subsided, but gradually returned, peaking that evening and night.

P5: “But then about an hour after getting back in the camp … I was vomiting. I was crawling all over the camp, literally trying to find somewhere cool to lie. I was really sick properly. Just vomiting up fluid. Literally just lay on the sand … vomiting, pulling sand over it, vomiting, pulling sand over it. and just desperately trying to find somewhere cool because it was just too hot in the tent and obviously I felt ill. And now I thought, and I felt so bad that I thought my race was done. I thought I was that ill, I thought I've not got the energy to line up the next day for 40 K”.

This highlights how physiological strain, including gastrointestinal distress and dehydration, was closely linked with psychological decline and reduced confidence in the ability to continue the race. In the case of a third individual, a combination of physical and mental decline led to his ultimate withdrawal from the event. He recounted experiencing extreme fatigue, nausea, and dizziness. In the preceding year, he had faced a similar predicament but recovered with the help of intravenous fluids supplied by the race medics, which enabled him to make a swift recovery and ultimately complete the race. Unfortunately, intravenous fluids were not offered to the participant at this year's event, resulting in a deterioration of his symptoms and his decision to abandon the race.

P1: “I just vomited everything up … probably sort of indicates heat stroke or something”.

This reflects how physical symptoms and environmental stressors can have the ability to impact both decision-making processes and race continuation. These insights demonstrate the disruptive nature of competing in a desert environment. Despite preparation and planning, participants remained vulnerable to the interplay of the heat, appetite loss, and GIS, suggesting that nutrition during the MdS and similar events is not simply about performance optimisation, but also adaptation to manage the body's breaking points in extreme conditions.

### Recovery as release and reset

This theme reflects recovery as both a physiological and psychological process, characterised by a shift away from structured control toward flexibility and relief following the demands of an event. The third and final interview centred on participants' approaches to the recovery period. Recovery was framed not only in terms of nutrition but also as a process of release, indulgence, and both physiological and psychological reset after the stress of the preparation and competition phases. Notably, participants' approaches were largely similar, with only minor variations reported.

In stark contrast to their pre- and in-race strategies, participants described adopting a relaxed and unstructured approach to eating during the recovery. Several participants spoke of indulging in foods that were typically excluded from their diets, including chocolate, crisps, and pizza. For some, this was accompanied by a feeling of freedom in allowing themselves to eat to their preferences without a simultaneous sense of guilt; they were “able to go to restaurants and eat food and eat pizza and carb up” (P1). Eating behaviours during this period were care- and thought-free, with indulging and an “eating whatever was straight in front of me” mindset (P5). This shift suggests that recovery was experienced not only as physical but also as psychological relief following prolonged restriction. Though there was an awareness of the deficits created by their recent endeavour, nutrition strategies to manage this were vague, with an overarching aim of eating more to replace carbohydrate stores. In a similar vein, others described letting go entirely of their nutrition plan and structure, instead eating instinctively. This was with the general aim of supporting weight regain and ensuring a high protein intake to support effective and timely recovery.

P2: “I ended up losing over the course of the period probably about seven and a half kilo, seven and a half kg of weight… just eating as I felt like I didn't have a set plan of I have to eat this or, anything… A lot of protein I would eat”.

Although participants, for the most part, exhibited similar attitudes towards the recovery period, subtle differences emerged. Most notably, the degree of lenience and dietary flexibility exhibited by participants varied. For one participant, though they recognised their approach was unstructured, they intended to only deviate slightly from their usual dietary pattern. After experiencing a severe bout of vomiting in the desert, her goal at this stage was to slowly return to regular portion sizes to avoid “overloading” her body:

P5: “tried to not overload my system, but at the same time I allowed myself to have whatever I wanted cause mostly what I was craving was vegetables and you know yoghurt and nuts and cheese and I don't, I wasn't craving anything awful anywhere … I did go to the sweet counter twice… but there's no there's, there's no, like, majorly structured strategy coming out the other end, apart from just don't overload myself”.

Comparably, while most participants regularly indulged throughout this period, one athlete swiftly returned to his regular dietary routine with only minor deviations to indulge.

P2: “So nothing drastically has changed. What I have changed is like I'll get a bag of Cheetos and cookies to kind of enjoy … . it's usually clean food and that I kind of consume on a daily basis, so not a drastic change so”.

These accounts highlight recovery as a necessary period to relinquish control, indulge, and both physiologically and psychologically reset. They underscored the importance of viewing recovery holistically, accounting for both physical and mental health when developing supportive recommendations and guidelines for this phase.

### Mental battles in the sahara

This theme captures the significant psychological strain experienced during the event, and how participants used various cognitive strategies to cope with fatigue, discomfort, and negative thoughts. Though not part of the initial aim of this research, it became evident from interviewing MdS participants that partaking in this event is often psychologically as well as physiologically challenging. For many participants, psychological challenges manifested as minor inconveniences such as dissatisfaction with the repetitive nature of their food flavours and textures, as well as boredom in the afternoons once the daily running stage had ended, feeling that they were “sitting around a lot with nothing to do” and “always thinking about food” (P1). Multiple participants reported that if they were to do this race again in the future, they would consider adding “a variety in their food” (P4) in the desert. Though “the food was fine” (P4), supplying the required energy and macronutrients for the event, eating the same products repeatedly became monotonous.

P5: “I think I would probably in terms of nutrition wise, try to have something more palatable in terms of more solid like a something that I could chew on … it wasn't a taste or the flavour of the food. It was the, the texture of food. I struggled because I would like something to chew on”.

Conversely, this event was accompanied by much more profound psychological challenges. For one participant, their mental state was described as being “in a hole” where he was putting in a lot of effort but “not moving particularly quickly” (P1). He recognised that he had potentially sacrificed too much food in order to keep his backpack as light as possible, forcing him into a severe energy deficit which may have exacerbated his suffering. His GIS, mainly nausea, vomiting, and reportedly heat-stroke symptoms, coupled with his psychological state, ultimately led to his withdrawal from the event.

P1: “I was in such a hole and such a bad state, both mentally and probably physically… that was it. I just pulled the plug on it”.

Another participant, despite having deliberately limited their social interactions in the build-up to the MdS, developed a stomach ailment at the beginning of the event. This illness resulted in complete appetite loss, and the participant discarded most of their pre-packaged foods. Consequently, this forced the participant to scavenge for food from others later in the event, significantly deviating from their original dietary strategy for the race. This same participant reported struggling with mental adversity as she persevered through the race, descending into a dark mental state characterised by negative thoughts. In order to distract herself from this negative headspace, she engaged herself in a variety of cognitive tasks, such as word association games.

P5: “trying to play mind games with myself like word, you know, word games like, you know, songs beginning with a through to z … Stupid things like words that rhymed with, you know. rhymed with X and just going literally trying to invent anything to keep my brain going. And at one point I had two songs going round in my head at the same time, which was like it was like some kind of torture because it was just felt so confusing. And then I was trying to separate them and, like, get one song out so I could just have the one song going on in my head”.

This illustrates how participants actively relied on cognitive strategies to manage fatigue, discomfort, and negative thoughts during prolonged exertion. She also experienced the psychological phenomenon of hallucinations, recounting two distinct separate incidents of this. Firstly, she heard two separate songs playing simultaneously in her mind, which she found extremely difficult to differentiate between. Additionally, despite consuming a hydration drink in a mixed-berry flavour, she vividly remembered the taste of coconut from this drink, starkly contrasting with the flavour she was drinking.

P5: “I was hallucinating that my water tasted like coconut water, which was amazing … considering it was, like, berry flavoured hydration tablets”.

This suggests that extreme physiological stress and fatigue may contribute to altered sensory perception and cognitive strain during ultra-endurance events. Despite this physiological and psychological state, this participant persevered and completed the race. These accounts reveal how the MdS tests athletes both physiologically and psychologically, with challenges ranging from food boredom and monotony to hallucinations and psychological collapse. These highlight the need to view ultra-endurance performance through a dual lens, recognising the importance of both physiological and psychological resilience in event success.

In summary, participants in the present study described an ongoing struggle to balance nutritional control with psychological wellbeing, as excessive control often led to stress and unhealthy behaviours. During ultra-endurance events such as the MdS, nutrition practices were strongly influenced by the desert environment, where managing hydration, salt intake, and gastrointestinal issues were central to maintaining performance. Despite advanced nutrition planning, many participants experienced GIS, fatigue, and illness, highlighting the unpredictable challenges of racing in such an extreme climate. Post-event recovery was characterised by relaxation, indulgence, and psychological release following prolonged discipline and rigidity. Finally, participants emphasised the profound psychological challenges of the MdS, from food monotony and appetite loss to hallucinations, illustrating that success in ultra-endurance events depends not only on physiological preparedness and nutrition strategies, but also on psychological adaptability and resilience under pressure. Overall, these findings highlight a close interaction between nutritional practices and psychological responses, with fatigue, gastrointestinal upset, and environmental stressors influencing both dietary behaviours and mental state throughout the event.

## Discussion

The present study aimed to qualitatively analyse the nutrition practices and experiences of five individuals participating in the MdS ultramarathon. Throughout this qualitative investigation, it became apparent that the research participants shared an array of distinct psychological traits and overarching behavioural patterns, notwithstanding a few exceptions. These findings may be partly explained by the interaction between physiological strain and psychological responses during prolonged exercise. Factors such as energy deficits, dehydration, and gastrointestinal distress are likely to contribute to increased fatigue and reduced cognitive capacity, which in turn may impair decision-making, motivation, and emotional regulation. From a theoretical perspective, this aligns with psychobiological models of fatigue, which emphasise the role of perceived effort and cognitive factors in endurance performance (36). This could influence athletes' decision-making, potentially impacting choices around pacing, hydration, and fuelling, resulting in reduced performance. This may also partly explain why several athletes described challenges relating to appetite, food intake, and negative thought patterns as the event progressed.

Previous qualitative analyses of MdS participants have typically taken the form of single-participant case studies (27, 31, 41). McCubbin et al. (26) examined the nutrition planning and intake of *n* = 5 MdS participants. However, this was a quantitative investigation. In contrast, the present study recruited *n* = 5 participants, the largest qualitative research of this cohort to date, providing a broader preliminary insight. In employing multiple interviews with each participant, this research aimed to capture athletes' broader approaches to the MdS event, encompassing their experiences in the preparation, participation, and recovery stages.

The participants in this investigation described their lifestyles as being anchored in routine while aiming to maintain a degree of flexibility. This flexibility was equally applicable to both their training routines and dietary practices. A core reasoning for this flexibility was to break from a past tendency of over-rigidity, which participants shared. This obsessive behaviour previously manifested itself through the rigid following of “fad” diets, elimination of entire food groups, and partaking in pro-longed bouts of fasting. This behavioural pattern became a source of stress in participants' lives and ultimately led to their under-fuelling for, and inadequate recovery from, their ultra-running activity. Compared to field sport athletes, long-distance runners have been previously observed to elicit significantly higher degrees of conscientiousness (42). Similarly, Roeh et al. (43) found marathon runners to be less disinhibited than sedentary individuals, a behaviour which may be exhibited through their ability to follow specific dietary regimens.

Ultra-endurance runners, due to their ability to complete sustained athletic endeavours and overcome extreme challenges in particularly demanding conditions, may also be characterised by their mental toughness. Originally believed to be prevalent solely amongst elite athletes, this trait is now recognised as existing in both elite and non-elite individuals (44). Some research has explored associations between mental toughness, high levels of physical activity, and broader personality constructs, including traits related to the “dark triad” (45). However, the relevance of these associations to MdS participants remains unclear, as previous research has not identified meaningful differences compared to the general population (32). Participants in this research also displayed autonomy in their training routines and, for the most part, trained and prepared for events alone, given the solo nature of the sport. Previous research has reported ultra-endurance runners as scoring low in affiliative extraversion, which encompasses social warmth, affectionateness, and a tendency to value close relationships, traits which may lend themselves to preferences for solo training (14). In terms of sourcing information to guide their nutrition practices, participants, though many reported previous discussions with nutrition professionals, placed a heavier reliance on their own experimentation over that of health professionals, a conclusion akin to previous findings (46).

Within this sample, the participants' nutrition strategies appeared to support their performance without evoking gastrointestinal discomfort. All participants reported having either experienced GIS themselves in the past or witnessed another athlete struggling with GIS. This is not surprising, given the high prevalence of GIS recorded in ultra-endurance running (47). While there exists a substantial degree of similarity in the behaviours and nutrition practices of participants, a notable individuality remains in relation to their experiences. This observation aligns with previous research findings, which highlight considerable diversity in the motivations and experiences of ultra-endurance runners (14). A narrative review conducted by Patryka and Waśkiewicz (18) further emphasises this diversity, stressing the complexity of motivations for endurance runners and the subsequent need for tailored methodologies in training and assistance to facilitate athletes in attaining their goals. Importantly, these findings suggest that nutritional challenges were not experienced in isolation but were closely intertwined with psychological responses, influencing how participants adapted their intake and overall race strategy.

A key similarity in participants' dietary approaches to the MdS event was their reliance on dehydrated foods throughout the race. These offer a source of lightweight nutrition which is easier to carry throughout the event than their hydrated counterparts. While these were not necessarily enjoyed by participants, they were recognised as a source of mental and physical discomfort that must be overcome to successfully complete the event. Ultra-endurance runners have previously reported that persistence and perseverance, as well as enduring and overcoming pain, are essential aspects of the mental toughness involved in ultra-endurance running (33). Participants of this research also had a shared awareness of the importance of hydration and salt intake to avoid hyponatremia, an issue commonly reported, especially at events with such challenging environmental conditions (48, 49). Diet observation research has previously found that athletes, in preparing for and competing in a 24 h endurance event, fail to meet carbohydrate recommendations, with their intake of this macronutrient having subsequent effects on their running performance (21).

Similar research investigating the experiences of five runners in preparation for and participation during the MdS has been more quantitative than the present study, focusing on athletes' nutritional planning and intake. However, some findings are akin to those of this research. These include a reported unpalatability of certain foods, usually those which are sweet, such as gels, or a loss of appetite due to unappealing food textures. Previous research into athletes experiencing ultra-endurance events has been quantitative in nature, monitoring variables such as body mass and percentage body fat throughout an athlete's training period and post-ultra-event (25).

Regarding the recovery after participating in an ultra-endurance event, this is an extremely physiologically and psychologically demanding process. Previous research has identified that it takes approximately 15 days for an athlete to return to their pre-race status after a 24 h ultra-endurance event (26). However, long-term effects relating to the recovery period have also been reported as existing up to one month after an ultra-event (50). Furthermore, multi-stage ultra-events have been shown to elicit more prolonged impairments in comparison to single-stage events, with the repetition of running at high intensities inducing longer-lasting peripheral fatigue than a single sustained bout of running. Recovery outcomes are influenced by an individual's training status, post-event activity, and nutrition strategies. Athletes should focus on meeting the current nutrition recommendations of 6–10 g.kg.day carbohydrates to restore glycogen stores and 1.2–2.0 g.kg.day protein to support muscle repair after an event. Additionally, for optimum rehydration athletes are recommended to weigh themselves before and after key sessions, then ingest a fluid volume greater than that lost (150%) while simultaneously ingesting 460 mg.L of sodium to restore water balance and avoid exercise-associated hyponatremia. Inadequate nutrition or hydration at this stage may interrupt the recovery process, ultimately delaying return to regular training (20). Ultra-endurance events can also lead to significant psychological stress, resulting in symptoms like increased fatigue and decreased cognitive function and vigour (14). The recovery approach of participants in this research was broadly similar in that it was relaxed and unstructured with no defined protocol, with only minor deviations to this approach reported. This approach allowed for both physical and mental reset, whereby athletes could take time away from the rigid dietary structures and prolonged discipline associated with competing. There is a complete dearth of scientific literature employing a qualitative design to investigate athletes' approaches to this stage of an ultra-endurance season (51).

Though not the initial aim of this research, it became apparent through conducting this investigation that the nutritional, physiological, and environmental demands of the MdS were closely intertwined with participants' psychological experiences throughout the event. Previous investigations into the psychological traits of ultra-endurance runners are sparse. As a result, a narrative review by Braschler et al. (52) on the personality traits of ultra-runners also included triathlon participants in their eligibility criteria, regarding every race longer than the typical 42.194 km marathon distance as “ultra”. A recent systematic review by Thuany et al. (53) reported that mental health issues are common among ultra-endurance runners, in particular eating disorders, exercise addiction, depressive symptoms, and sleep disturbances. The findings of this study suggest that psychological factors play an important role in these participants' experiences of ultra-endurance events. However, further high-quality research is necessary to gain insight into the factors underlying these issues, how they impact performance, and how they are managed. For participants in this research, there was a shared psychological challenge of boredom, which they had to overcome due to free time after each day's running or the repetitive, monotonous flavours and textures of their dehydrated foods. This often led to participants losing their appetite and having a slight aversion to their meals, eating them solely for their nutritional value. The repetitive nature of these foods, combined with the desert heat and prolonged exertion could have collectively made it challenging for athletes to consistently maintain adequate energy intake throughout the event. In a qualitative investigation into ultra-endurance runners' experiences participating in a 125 km ultra-marathon, similar issues arose with participants, who experienced GIS, physical pains, and thoughts about quitting. Akin to the participants in this research, multiple athletes also described experiencing a mental and physical battle during the event (32). This may help to explain why participants described entering negative mental states or experiencing difficulty maintaining motivation despite prior preparation.

Certain participants in this research confronted markedly more profound physiological and psychological challenges, including experiencing hallucinations, particularly dark thoughts, and debilitating physical pains, including GIS. For the psychological challenges specifically, mental games were a strategy used to pass time and focus elsewhere than on their negative thoughts. Previous research into individuals' experiences of an ultra-marathon has also noted that athletes use coping strategies such as making small goals or engaging in mental battles to overcome stressors (32). These findings support the call for sport psychology to increase its engagement with ultra-endurance runners and investigate how these individuals deal with and develop the abilities to manage stressors throughout their training and racing; a call already made in previous research (30).

### Limitations

Due to the small sample size in this study, its findings should be considered exploratory in nature. Though this research offers valuable insight into the individual experiences of athletes participating in such unique and challenging events, they are not generalisable to the broader ultra-marathon running or ultra-endurance sports populations. Nonetheless, the results of this study may be used to guide and inform the focus of future qualitative investigations into ultra-endurance runners’ experiences. To develop a more comprehensive understanding of the shared experiences and common challenges throughout the majority of this population, future research should aim to include larger sample sizes. Additional limitations include the relatively short duration of interviews, which, although conducted at multiple timepoints, may have limited the depth of some responses. The study also relied on self-reported data, which may be subject to recall bias or social desirability bias. Furthermore, while reflexive practices were implemented throughout the research process, the potential influence of researcher interpretation cannot be entirely eliminated. As with qualitative research, the findings are not intended to be generalisable but may offer transferable insights to similar ultra-endurance populations and contexts.

### Practical implications

The findings of this research hold several practical implications, both for ultra-endurance runners and those supporting them. Although further research is needed to refine evidence-based approaches in ultra-endurance running, this study offers practical insights for athletes and professionals supporting them until formal guidelines are established by sport nutrition organisations.

#### For athletes and coaches

In relation to day-to-day nutrition, it seems that these athletes benefit from individualised and flexible strategies that balance control with adaptability. In preparing for events, nutrition professionals should encourage pre-race mock weeks whereby athletes trial their race day nutrition protocol, which in this instance would involve a heavy reliance on dehydrated meals, energy gels, and electrolyte sources such as stock cubes. This may help to identify foods which are energy-dense, lightweight, and well-tolerated during competition. Additionally, experimenting with a range of flavours and textures may support athletes in avoiding food monotony during an event, which can result in a lower food or hydration intake, ultimately impairing performance.

#### For practitioners

Regarding developing protocols to support exercise recovery, both the physiological and psychological toll of competing in an ultramarathon should be recognised. Post-event, professionals should consider the psychological relaxation needs of their athletes and work to balance a targeted micronutrient intake with a flexible nutrition approach. Finally, psychological resilience emerged as a critical factor for completing this event. Participants described using strategies such as distraction techniques, mind games, and self-talk to manage fatigue, boredom, and negative thoughts, suggesting that preparing these strategies in advance may support athletes during prolonged exercise.

## Conclusion

The present study aimed to qualitatively analyse the nutrition practices and experiences of five individuals participating in the MdS ultramarathon, offering insight into the multifaceted demands of ultra-endurance running. Participants emphasised the importance of individualised nutrition and hydration strategies developed through prior planning and experimentation, aiming to balance energy density, palatability, and gastrointestinal comfort. Nutrition choices were shaped by the desert environment and the self-sufficient nature of the event, heavily comprising dehydrated foods, energy bars and gels, and electrolyte supplements. Participants also reported significant psychological strain, from monotony and appetite loss to distressing thoughts and hallucinations, suggesting that ultramarathon performance is influenced by both physical and psychological adaptability. Overall, this research highlights the interaction between nutritional, physiological, and psychological demands in multi-stage ultramarathons such as the MdS. These findings provide context-specific insight into how athletes experience and respond to the combined demands of such events. Future research should aim to further explore the interaction between nutritional and psychological factors in ultra-endurance settings, particularly through the development of integrated support strategies that combine nutritional and psychological interventions.

## Data Availability

The original contributions presented in the study are included in the article/Supplementary Material, further inquiries can be directed to the corresponding author/s.
